# Multiple Sclerosis Following SARS-CoV-2 Infection: A Case Report and Literature Review

**DOI:** 10.7759/cureus.19036

**Published:** 2021-10-25

**Authors:** Sobia Sarwar, Sylvette Rogers, Alaa S Mohamed, Enitare Ogula, Rihanat A Ayantayo, Ahmed Ahmed, Iram Shahzadi, Saurabh Kataria, Romil Singh

**Affiliations:** 1 Neurology, Independent Medical College, Faisalabad, PAK; 2 Family Medicine, Caribbean Medical University, Des Plaines, USA; 3 Neurology, Augusta University, Augusta, USA; 4 Medicine, Saint James School of Medicine, Park Ridge, USA; 5 Internal Medicine, Nigeria Police Medical Center, Ondo, NGA; 6 Neurology, Mayo Clinic, Rochester, USA; 7 Internal Medicine, Rawalpindi Medical University, Rawalpindi, PAK; 8 Neurology, Ochsner Louisiana State University Health Sciences Center, Shreveport, USA; 9 Clinical Observation and Research, Neurology and Neurocritical Care, University of Missouri Health Care, Columbia, USA; 10 Distant Research, Neurology, West Virginia University, Morgantown, USA; 11 Critical Care, Mayo Clinic, Rochester, USA

**Keywords:** demyelinating diseases, post-covid complications, multiple sclerosis, sars-cov-2, covid-19

## Abstract

Coronavirus disease 19 (COVID-19) is caused by severe acute respiratory coronavirus 2 (SARS-CoV-2). Apart from respiratory manifestations, COVID-19 can affect the nervous system due to its neurotropic features. Neurological manifestations and complications include headache, polyneuropathies, cerebrovascular accidents, seizures, encephalopathy, and demyelinating disease. We describe a case of multiple sclerosis, a demyelinating disease following COVID-19 infection, rarely reported in the literature.

A 47-year-old female presented with fatigue, blurry vision, numbness, and signs of upper motor neuron lesions that had occurred three weeks after COVID-19 infection. Magnetic resonance imaging of the brain revealed demyelinating lesions in the periventricular area of both hemispheres, suggesting a demyelinating disease. A provisional diagnosis of multiple sclerosis was made. Her condition improved after the commencement of methylprednisolone.

## Introduction

Coronavirus disease 19 (COVID-19) is caused by severe acute respiratory coronavirus 2 (SARS-CoV-2), which typically manifests with fever, myalgia, and signs and symptoms of the respiratory system and causes substantial morbidity and mortality in severe cases such as multiorgan failure and acute respiratory distress syndrome [1-4]. The novel coronavirus is also a neurotropic virus causing complications in both the central and peripheral nervous systems, particularly during the post-recovery phase with a prevalence of 36% after a viral infection, and those complications include polyneuropathies, headache, seizures, ataxia, cerebrovascular diseases, and demyelinating diseases [5-6]. Demyelinating disorder, such as multiple sclerosis following a COVID-19 infection, has also been reported in the literature [7]. Herein, we describe a rare case of multiple sclerosis in a female following SARS-CoV-2 infection.

## Case presentation

A 47-year-old female with a past medical history of diabetes mellitus came to an outpatient clinic for post-COVID follow-up. She complained of severe fatigue throughout the day for the last six days, associated with intermittent tingling and numbness on the left side of the leg and arm. She had blurry vision in her left eye for the last 18 hours associated with intermittent mild pain behind the left eye exacerbated by eye movement. She had not experienced these symptoms before. She denied any history of migraines and any family history of neurological disease. She was compliant with her diabetic medication.

She was admitted to the hospital three weeks back due to a COVID-19 infection with typical signs and symptoms, including fever, dyspnea, cough, and myalgia. She was treated with remdesivir, dexamethasone, and supplemental oxygen due to fluctuation in oxygen levels. Her symptoms gradually improved, and she was discharged four days later on dexamethasone and antipyretics with isolation instructions.

On examination, the patient looked anxious, anicteric, and well-oriented in time, place, and person. She was afebrile, with a blood pressure of 110/80 mmHg, respiratory rate of 21/minute, heart rate of 92/minute, and oxygen saturation of 97%. On neurological exam, her visual acuity was 60/200 in her left eye, with no visual problem in her right eye. A relative afferent pupillary defect was noted in the left eye, and fundoscopic examination showed mild inflammation of the optic disc. Other cranial nerves were intact, with no signs of meningeal irritation. Hyperreflexia was noted in the lower limbs, predominantly on the left side, along with ipsilateral clonus. Numbness was noted in the upper limb with mild sensory loss in a glove and stocking pattern. She also had reduced incitement and a delayed thought process. Respiratory and cardiovascular examinations were non-significant.

**Figure 1:**
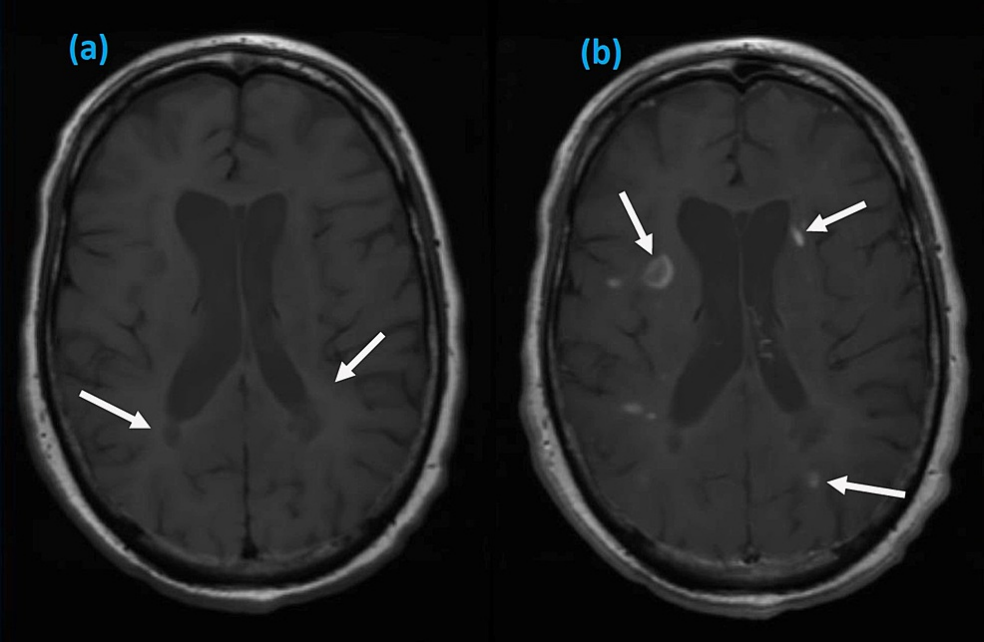
Axial T1 MRI images showing periventricular lesions of various intensities in posterior (a, b) and parietal lobe (b) MRI: magnetic resonance imaging

**Figure 2:**
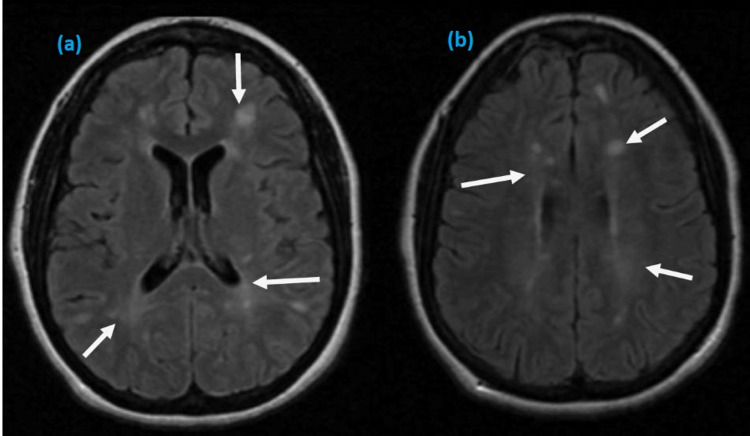
Axial T2 MRI images showing hyperintense signals in both hemispheres (a, b). MRI: Magnetic resonance imaging

Initial serum investigations were within normal range except for mild elevation of erythrocyte sedimentation rate. Her repeat SARS-CoV-2 test was negative; however, serology was positive for COVID-19 immunoglobulin G (IgG) and IgM. Magnetic resonance imaging (MRI) of the brain was performed and revealed multiple scattered periventricular lesions with contrast enhancement in T1-weighted axial images (Figure 1) and hyperintense lesions involving periventricular areas of both hemispheres on T2-weighted images (Figure 2). MRI spine was normal. On further investigations, viral serologies for syphilis, hepatitis B and C, and human immune deficiency virus were negative. Autoimmune screening, including antineutrophil cytoplasmic antibodies and other cytoplasmic antibodies, was non-reactive. She did not give consent for a lumbar puncture.

Based on history, physical examination, and detailed investigations, a diagnosis of multiple sclerosis triggered by COVID-19 was made, as no other etiology was identified. She was managed with 1 g of prednisolone daily and symptomatic treatment. Her vision improved gradually, and she also reported improvement in her fatigue and other neurological symptoms. She was discharged on tapering oral dexamethasone with follow-up.

## Discussion

COVID-19 mainly affects the respiratory system, and common respiratory manifestations include fever, dyspnea, cough, and sore throat. Gastrointestinal and cardiovascular complications include diarrhea, nausea, acute pancreatitis, myocardial infarction, and acute coronary syndrome [1]. Neurological complications have also been reported, and common manifestations include headache, anosmia, cerebrovascular disease, and seizures [5]. The proposed pathophysiology of multisystem involvement in COVID-19 is due to the presence of angiotensin-converting enzyme 2 (ACE2) receptors in multiple organ systems and having a high affinity for the SARS-CoV-2 virus [7]. Apart from other neurological manifestations, demyelinating disease, such as multiple sclerosis has not been reported widely due to COVID-19 infection. We have reported cases of multiple sclerosis after COVID-19 in Table 1.

**Table 1:** Multiple sclerosis due to SARS-CoV-2 infection MS: multiple sclerosis, ADEM: acute disseminated encephalomyelitis, N/R: not reported

Author	Age (year)	Sex	First symptom after COVID-19 infection (weeks)	Demyelinating disease	Major manifestation	Type of Demyelinating disease	Management
Palao et al. [8]	29	Female	3	Yes	Optic neuritis, anosmia	MS	Methylprednisolone
Yavari et al. [9]	24	Female	4	Yes	Diplopia, paresthesia	MS	Interferon-beta
Ismail et al. [10]	36	Male	8	Yes	Ataxia, vertigo	MS	Methylprednisolone
Moore et al. [11]	28	Male	2	Yes	Vertigo, blurry vision	MS	Methylprednisolone
Zanin et al. [12]	54	Female	N/R	Yes	Unconsciousness	MS	Dexamethasone
Karsidag et al. [13]	18	Female	2	Yes	Dizziness, Vertigo	MS	Methylprednisolone
Karsidag et al. [13]	42	Female	4	Yes	Jaw and facial pain	MS/ADEM	Methylprednisolone
Karsidag et al. [13]	32	Male	16	Yes	Numbness, weakness in the right leg	MS/ADEM	Methylprednisolone

The neurotropic properties of COVID-19 are due to the direct invasion of SARS-CoV-2 through the nasopharyngeal area into the brain, the presence of ACE2 receptors in the nervous system, including glial cells, and basal ganglia and their affinity to SARS-CoV-2 resulting in the development of symptoms [14]. A severe inflammatory response against SARS-CoV-2 results in the production of inflammatory and pro-inflammatory mediators and cytokines. These inflammatory mediators disrupt the blood-brain barrier and induce glial cells activation and demyelination of nerve fibers [15]. Molecular mimicry, such as the production of autoantibodies induced by cytokine storm, can also result in immune-mediated injury to nervous tissues. Microinfarction and cerebral ischemia may also result from microangiopathy induced by complement activation and cytokine response [14-15]. Several studies have also linked autoimmune diseases with many viruses, such as parainfluenza virus and coronavirus, after six to seven weeks of exposure [16].

Multiple sclerosis is a nervous system autoimmune disease with a prevalence of 0.91 million in the USA [17]. Multiple sclerosis is a demyelinating disorder affecting the brain and spinal cord with a wide range of clinical manifestations, which differ significantly from person to person and over the course of the illness depending upon the lesion location. Manifestations include visual problems, upper or lower motor neuron signs, fatigue, numbness, ataxia, body aches, and bowel, bladder, or sexual dysfunction [18]. Multiple sclerosis is diagnosed using McDonald's criteria in patients between 20 to 50 years old [19]. Other than infectious etiology, genetic and geographical etiologies may also play a role in the pathogenesis of multiple sclerosis [20]. Therefore, the treatment of multiple sclerosis is tailored to symptomatic management and the use of immunomodulatory disease-modifying agents [18].

Since our patient met the criteria for the diagnosis of multiple sclerosis based on signs and symptoms separated in space and MRI findings. It can be assumed that the pathogenic process of multiple sclerosis had already begun before SARS-CoV-2 infection due to geographical, environmental, and genetic factors. Thus, in our case, COVID-19 might have appeared as a precipitating or triggering factor rather than being a direct consequence of infection itself. However, triggering and exacerbating autoimmune diseases in a patient with COVID-19 is increasing in the literature, and the causal association between multiple sclerosis and COVID-19 infection is warranted from future studies.

## Conclusions

SARS-CoV-2, with its neurotropic characteristics, could trigger neurological auto-immunity and might appear in the place of neurological disorders. Our case highlights a possible role of SARS-CoV-2 in the pathogenesis of multiple sclerosis; however, our study is limited to a single case. Further evidence from future studies is warranted to establish the causality, and more cases of demyelinating diseases are required to affirm themselves after SARS-CoV-2.
